# Computational Assessment of Thermokinetics and Associated Microstructural Evolution in Laser Powder Bed Fusion Manufacturing of Ti6Al4V Alloy

**DOI:** 10.1038/s41598-020-63281-4

**Published:** 2020-05-05

**Authors:** Mangesh V. Pantawane, Yee-Hsien Ho, Sameehan S. Joshi, Narendra B. Dahotre

**Affiliations:** 1 Laboratory for Laser Aided Additive and Subtractive Manufacturing, Department of Materials Science and Engineering, University of North Texas, 1155 Union Circle-305310, Denton, TX 76203-5017 USA; 2 Center for Agile and Adaptive Additive Manufacturing, University of North Texas, Denton, TX 76207 USA; 3 Present Address: Cummins, Inc., Columbus, Indiana USA

**Keywords:** Scanning electron microscopy, Computational methods, Metals and alloys

## Abstract

Although most of the near non-equilibrium microstructures of alloys produced by laser powder bed fusion (LPBF) additive manufacturing (AM) are being reported at a rapid rate, the accountable thermokinetics of the entire process have rarely been studied. In order to exploit the versatility of this AM process for the desired properties of built material, it is crucial to understand the thermokinetics associated with the process. In light of this, a three-dimensional thermokinetic model based on the finite element method was developed to correlate with the microstructure evolved in additively manufactured Ti6Al4V alloy. The computational model yielded the thermal patterns experienced at given location while building a single layer through multiple laser scans and a whole part through multiple layers above it. X-ray analysis of the resultant microstructure confirmed the presence of acicular martensitic (*α*′) phase of (002) texture within the build-plane. Computationally predicted magnitude of the thermal gradients within the additively manufactured Ti6Al4V alloy in different directions (X, Y, and Z) facilitated the understanding about the evolution of grain morphology and orientation of acicular martensite in prior *β* grains. The scanning electron microscopy observations of the alloy revealed the distinct morphology of phase precipitated within the martensitic phase, whose existence was, in turn, understood through predicted thermal history.

## Introduction

Laser powder bed fusion (LPBF) alternatively known as selective laser melting (SLM) is one of the rapidly evolving additive manufacturing (AM) techniques. LPBF process offers various advantages over conventional subtractive manufacturing techniques as it overcomes the critical issues of design intricacies, material and cost efficiency, and the surface finish of the product^1,2^. On the contrary, this layer-by-layer additive manufacturing process combined with a rapid rastering laser source to fuse the powder particles renders spatially varying complex thermokinetics and fluid dynamics^3^. Such a thermal process yields an entirely different near non-equilibrium microstructure compared to that obtained with conventional manufacturing processes. However, due to lack of in-depth understanding of this process in terms of thermokinetics (thermodynamics and kinetics), the physical metallurgy of various alloys evolved during LPBF process remains in its infancy. Although the development of *in*-*situ* probing of the process is underway, if not possible, it is challenging to achieve the essential sensitivity of data to be monitored due to aspects including but not limited to (1) extremely high thermokinetics (high temperature, and high heating/cooling rates), (2) very small laser-material interaction region, and (3) obstruction due to development of material plume and/or plasma in the laser-material interaction region to be probed.

The computational efforts to conceive the dynamics of the LPBF process through the finite element modeling (FEM) have been improving consistently^4–10^. A majority of FEM efforts have been focused on developing the understanding of the fluid dynamics during laser-material interaction to optimize the laser based AM process parameters for sound fabrication of the parts^4–6^. However, thermokinetics, thermal history, and their direct correlation with the evolution of thermal stresses and microstructure during LPBF process have been sparsely studied to understand the evolution of the microstructure. In LPBF process, typically the laser due to its small beam diameter (50 µm–600 µm) interacts with very small region (0.002 mm^2^–0.28 mm^2^) of the powder bed which in turn leads to very high power density (>${10}^{4}$ W/mm^2^). Such high power density in an extremely small laser-material interaction region yields very high heating rate followed by rapid cooling (${10}^{4}$ K/s–${10}^{7}$ K/s). Furthermore, as this process produces a three-dimensional product, the thermokinetics vary in different directions within an individual layer and also across build layers of the product. With such an inherently complex thermokinetics associated with the process, each region in the LPBF product experiences a distinct and unique thermal signature/history. These thermal signatures manifest into multiple melting, annealing, and tempering heat treatments, thereby generating uniquely different microstructure at various locations of the product. These thermal signatures/histories (cycles) are influenced by the process parameters and thermophysical properties of the material. Thus, in depth understanding of effects of these parameters on the thermal history experienced by the LPBF product is essential. Such insight is likely to allow monitoring of thermokinetics and resulting microstructure during LPBF process.

In light of the scenario mentioned above, the current work presents a three-dimensional computational thermal model based on the finite element method to simulate the thermokinetics of the LPBF process. The study involves a computationally efficient approach of adaptive re-meshing and individual study steps corresponding to building of each layer. Such approach allows accurate consideration of the effect of heat build-up within a laser track and consequently within each layer and across built structure. Furthermore, the computational model was validated with characterization of microstructure evolved during LPBF printed Ti6Al4V alloy. Ti6Al4V is often employed in a variety of fields including but not limited to biomedical, energy, marine, and defense applications, owing to its excellent set of mechanical and chemical properties. Conventionally manufactured Ti6Al4V alloy shows a mixture of $\alpha$ (HCP) and $\beta$ (BCC) phases^11^, whereas the LPBF manufactured parts, mostly result in near non-equilibrium accicular martensitic structure^12–15^. However, the evolution of particularly unique microstructure and its correlation to thermokinetics associated with LPBF process have not been addressed yet at sufficient depth and understanding. In light of this, this research aims at bridging this gap through the approach of computational assessment of thermokinetics and associated microstructural evolution in laser powder bed fusion additively manufactured Ti6Al4V alloy.

## Materials and Methods

**Figure 1:**
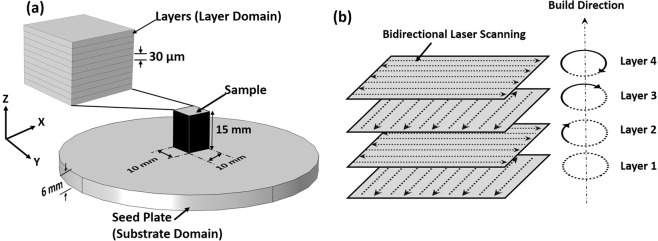
Schematic representation of (**a**) various domains considered in the model and (**b**) laser beam scanning and layer orientation strategy.

The LPBF parts were produced in the AconityMIDI system equipped with continuous wave (CW) Nd:YAG laser ($\lambda$ = 1070 nm) of 85 µm beam diameter (D). The powder bed was prepared with a commercial grade-23 (received from Carpenter Additive) Ti6Al4V alloy of extra-low interstitial elements. Ti6Al4V powder particles were in the range of 15 µm–45 µm. The blocks (10 mm x 10 mm x 15 mm) of Ti6Al4V were additively manufactured by multiple laser fused powder layers on a Ti6Al4V circular seed plate of 100 mm diameter and 6 mm thickness as schematically presented in Fig. 1a. The samples were produced using laser power ($P$) of 150 W and a scanning speed ($v$) of 800 mm/s. The activity of oxygen in the processing chamber was maintained below 50 ppm by continuous purging of Ar gas. Each fused powder layer in turn was fabricated by running multiple subsequent parallel linear laser scans. The two consecutive linear laser scans were made in reverse directions (bidirectional scanning) for the purpose of maintaining continuity in laser processing as schematically depicted in Fig. 1b. The distance between the centers of two consecutive laser scans, which is referred here as hatch spacing ($h$), was maintained at 120 µm. Each powder layer thickness ($d$) was kept constant at 30 µm (Fig. 1a) and the orientation of bi-directional laser scanning in each layer was rotated through 90 degrees with respect to the previous layer as depicted in Fig. 1b. A delay between the building of two consecutive layers, termed as an interlayer delay time (${t}_{i}$) was kept as 16 s. Thus, under the processing scheme adopted in the present study and described above an average input laser energy per unit volume or energy density ($E$) and a laser residence time (${t}_{r}$), which is the time of interaction of a laser beam at a given location during scanning, are represented by the following Eqs. 1 and 2 respectively.

1 $$E=\frac{P}{v\times h\times d}$$

2 $${t}_{r}=\frac{D}{v}$$

Based on the processing parameters employed, the energy density (E) was 52.08 J/mm^3^ and laser residence time (${t}_{r}$) was 106.25 μs. The processing scheme and associated operational parameters used in the present study were chosen based on extensive prior experience of the authors in the area of laser-based materials processing/manufacturing that facilitated physically/mechanically sound (pore and crack free) fabrication of the parts.

The LPBF blocks were cut parallel to the YZ plane (Fig. 1a) for microstructural observations along the layer build direction (Z). The samples were polished on SiC paper sequentially up to 4000 grit size, followed by lapping in silica suspension. The samples were further electropolished at 5 °C in electrolytic solution consisting of 90 ml distilled water, 730 ml ethanol, 100 ml ethylene glycol monobutyl ether, and 78 ml perchloric acid. The electropolished samples were exposed to Kroll’s etchant for 3 seconds to delineate the microstructural features. The microstructural examination was performed on the nano scanning electron microscope (SEM) by FEI. In addition, the semiquantitative elemental analysis was performed using energy dispersive spectroscopy (EDS). The samples were also examined in the XY plane, orthogonal to the build direction (Fig. 1a), to detect the presence of any phases by Rigaku Ultima high-resolution X-ray diffractometer (XRD). The X-ray diffractometer was operated with Cu-K$\alpha$ source ($\lambda$ = 1.54 Å), step size of 0.025°, and scanning speed of 1°/min in a range of 20–90°. Simultaneously, conventionally manufactured Ti6Al4V alloy plate was also examined with XRD for comparison of phases in conventionally and LPBF manufactured samples.

## Numerical Modeling

A computational thermal model was developed and simulated using the finite element method (FEM) on the COMSOL platform. The design of the model involved two unified domains: the disc-shaped domain to account for the seed plate (100 mm diameter × 6 mm thickness); and the cuboid (10 mm × 10 mm × 0.03 mm) for the individual layer domain of the complete part (10 mm × 10 mm × 15 mm) that was additively printed during LPBF process (Fig. 1a). Subsequent layer domains were added on previous ones after interlayer delay time (${t}_{r}$) of 16 s through distinct study step. In order to account for heat content of the previously build layer, the model configuration was modified in COMSOL solver. Accordingly, the model configuration was modified in each study step to locate the moving laser beam accurately as the layers are built. The substrate domain was meshed with a relatively coarser element size than the layer domain. The finer meshing of the layer domain was kept to a maximum element size of 0.08 mm. The computation was accurately facilitated by applying adaptive meshing refinement to the layer domain.

The computational model incorporates temperature-dependent thermophysical properties of the Ti6Al4V alloy^8,16,17^ to accurately display the effect of evaporative heat flux, phase change, and thermokinetic output. The laser processing parameters listed earlier were input in the model to obtain the corresponding thermokinetic outcome. In order to compute the model by taking into account various physical phenomena occurring during the laser-material interaction, a continuum layer domain with isotropic thermophysical and mechanical properties was assumed. Additionally, the model considered Gaussian laser heat flux distribution ($TE{M}_{00}$ mode).

### Governing equations

The top surface (XY) of the layer domain was exposed to the moving laser heat flux ${Q}_{(x,y)}$ with the Gaussian profile, which can be expressed as per Eq. 3.

3 $$Q(x,y)=P(1-R).\frac{{A}_{abs}}{\pi {s}^{2}}\,\exp \,\left[\,-\,\frac{{(x-x{\prime} )}^{2}+{(y-y{\prime} )}^{2}}{2{s}^{2}}\right]$$

where ${A}_{abs}$ is the absorptivity (0.45), R is the reflectivity (0.09), $s$ is the standard deviation of laser heat distribution in both x and y direction in XY plane. In the current study, s = 0.0425 mm. *x*′ and *y*′ represents the co-ordinates of the moving laser beam on XY geometric frame. These can be further expressed as Eqs. 4 and 5 respectively.

4 $$x{\prime} =({x}_{0}\pm nh\,\sin \,\theta )\pm (v\,\sin \,\theta )t$$

5 $$y{\prime} =({y}_{0}\pm nh\,\cos \,\theta )\pm (v\,\cos \,\theta )t$$

In above equations (Eqs. 4 and 5), $n$ represents number of the laser track ($n$ = 1, 2, 3, 4, ...), $\theta$ is the angle at which orientation of laser scanning is aligned in a layer. For example, in current study, $\theta$ is either 0° or 90° alternatively in each layer. ${x}_{0}$ and ${y}_{0}$ can be initial or final coordinates of a single laser track. These coordinates switch alternatively with each consecutive track to follow a bidrectional scanning pattern. For example, in this model, for $\theta$ = 0°, the coordinates of ${x}_{0}$ and ${y}_{0}$ switch from initial position (0, 0) to final position (0, 10) with each laser track. For unidrectional scanning pattern, coordinates of ${x}_{0}$ and ${y}_{0}$ will be constant.

The heat received from the laser source is conducted through the material domain, which is partially lost via convection and radiation. Additional heat loss occurs above the vaporization temperature through evaporative heat flux (${Q}_{evp}$) as the heat is carried away by vapors. Thus, total heat flux can be given as Eq. 6

6 $$-\,k\nabla T=Q(x,y)-{Q}_{evp}-h[T-{T}_{amb}]-\epsilon \sigma [{T}^{4}-{T}_{amb}^{4}]$$

where T is the temperature (K), $k$ is the thermal conductivity (W m^−1^ K), $\epsilon$ is emissivity, *σ* is the Stefan–Boltzmann constant ($5.67\times {10}^{-8}\,{\rm{W}}/{{\rm{m}}}^{2}{{\rm{K}}}^{4}$), *h* is the convective heat transfer coefficient (20 W/(m^2^K)), and ${T}_{amb}$ is the ambient temperature (300 K)^8,16,17^.

The other major governing equations involved the Navier-Stokes equation (Eq. 7) and the Fouriers law (Eq. 8). The former (Eq. 7) provides the velocity distribution of the molten alloy considering its dynamic viscosity ($\mu$) and buoyancy force (${F}_{B}$). The latter offers transient temperature distribution inside the domain (Eq. 8). These equations were solved together in the model with the continuity equation (Eq. 9).

7 $$\rho {C}_{p}\frac{\partial T}{\partial t}+\rho {C}_{p}(\overrightarrow{u}.\nabla T)=\nabla .(k\nabla T)$$

8 $${\rho }_{0}\left[\frac{\partial \overrightarrow{u}}{\partial t}+\overrightarrow{u}.(\nabla \overrightarrow{u})\right]=\nabla .[\,-\,pI+\mu (\nabla \overrightarrow{u})+(\nabla \overrightarrow{u}+(\nabla {\overrightarrow{u}}^{T}))]+{\overrightarrow{F}}_{B}$$

9 $$\rho \nabla .(\overrightarrow{u})=0$$

In above equations $\rho$ is the density of the material (kg m^−3^), p is the pressure (N m^−2^), $I$ is the identity matrix, ${C}_{p}$ is the specific heat of the material (J kg^−1^ K), $\overrightarrow{u}$ is the fluid velocity (m/s), and $\mu$ is the dynamic viscosity^8,16,17^. Further additional equations corresponding to fluid dynamics and the details related to computational modeling of laser material-interaction during laser-based materials processing and manufacturing were adopted from earlier work by the authors^18–20^.

## Results and Discussion

### Thermokinetic assessment

#### Thermal Evolution in Single Laser Track

**Figure 2:**
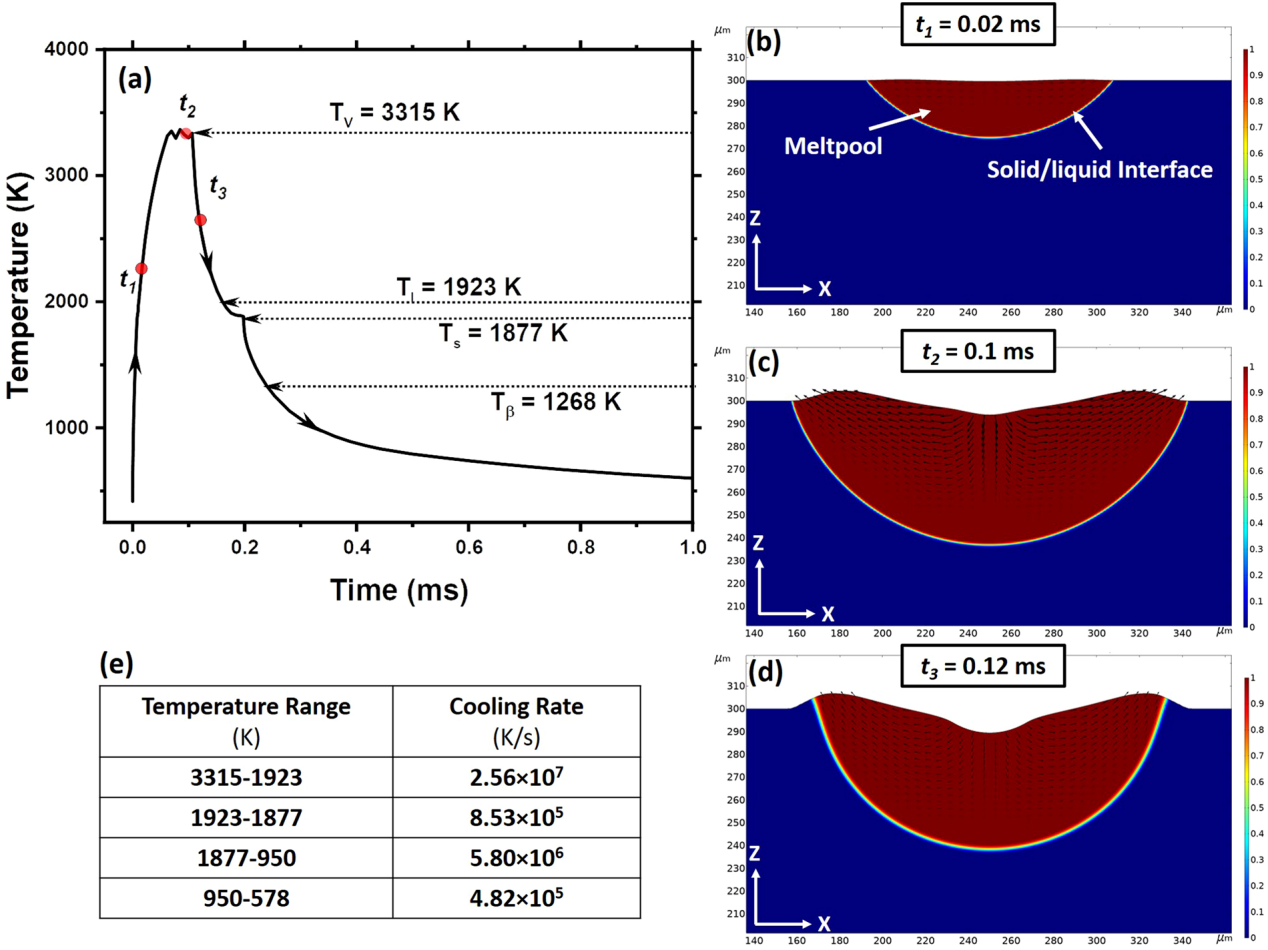
Computationally predicted (**a**) time-temperature relationship in a single laser track and (**b**–**d**) simulated event meltpool evolution at specific times in YZ plane with (**e**) predicted cooling rates within temperature ranges.

The fundamental understanding of the localized thermokinetic behavior of Ti6Al4V during LPBF process was developed by simulating a single laser track in a material domain with the laser parameters ($P$ = 150 W, $v$ = 800 mm/s) specified earlier. The surface temperature at specific location within a single laser track was computationally derived as function of time and presented in Fig. 2a. Corresponding meltpool evolution as function of time was simulated in the YZ plane while the laser beam was traveling along X direction (Fig. 1a). These simulations at three distinct events ${t}_{1}$ (0.02 ms), ${t}_{2}$ (0.1 ms), and ${t}_{3}$ (0.12 ms) are presented in Fig. 2b–d, respectively. These three specific times were elected for computational simulations because under the set of laser processing parameters adopted in the present study, all these times fall within the thermodynamic conditions where the laser-interaction zone is entirely in molten (during heating), molten+vapor (during heating), and molten (during cooling) states. Observation of the material during these transitions is especially important as it tends to develop specific surface morphologies through these transitions as explained in the following description.

The surface temperature rapidly rises at the heating rate of 6.71 × 10^7^ K/s and develops a meltpool (Fig. 2b). As this meltpool evolves, the fluid experiences the Marangoni effect, gravity forces, buoyancy effect, and curvature effect due to spatially varying temperature, viscosity, surface tension, and density within the meltpool (Fig. 2c). The hydrodynamics of the meltpool can be realized through the arrows, which indicate its velocity field (Fig. 2b–d). At the vaporization temperature (3315 K), while the meltpool loses some material as vapors, these vapors generates recoil pressure, thereby further influencing the meltpool dynamics (Fig. 2c). At this stage, the heat is lost due to the evaporative cooling effect (evaporative heat flux loss); besides, the recoil pressure also influences vaporization temperature^18,21^. Consequently, the surface temperature fluctuates around the vaporization temperature (Fig. 2a). The surface deformities induced by above-mentioned complex hydrodynamics continues to evolve past time ${t}_{3}$ (Fig. 2d) as the temperature plummets drastically at a rapid rate of 1.88 × 10^7^ K/s till the solidus temperature ${T}_{s}$ (1877 K) of T6Al4V. This physical morphology of the surface is retained only until it is disturbed/covered by the incoming layer. However, the surface morphology generated through such complex hydrodynamics on the final layer determines the final surface finish of the component produced using LPBF process. Hence, it is extremely important to thoroughly understand and control the hydrodynamics of the process to achieve desired surface finish.

In addition to casting a light on the hydrodynamic behavior of material at high temperatures, the computationally predicted time-temperature relationship also provides signatures of states of material while experiencing rapid temperature drop. The predicted time-temperature relationship indicated varying cooling behavior before it reaches 578 K at the end of 1 ms (Fig. 2a). The cooling rates within specific temperature ranges are listed in the table given in Fig. 2e. The cooling rate of the meltpool (2.56 × 10^7^ K/s) is highest in temperature range of 3315 K–1923 K. A gradual change in the slope of time-temperature plot (8.53 × 10^5^ K/s) corresponding to the temperature zone of 1923 K–1877 K is due to evolution of the latent heat associated with the mushy state of the alloy (Fig. 2a). The cooling rate decreases further nearly by order of magnitude (5.80 × 10^6^ K/s) over the temperature range of 1877–950 K, and thereafter it continues to drop further (4.82 × 10^5^ K/s) till the beginning of next laser track. This varying cooling rate within a single time-temperature cycle is due to temperature-dependent thermophysical properties of the alloy and also the process and material parameters such as but not limited to laser power, scanning speed, powder particle shape and size, seed plate, size and shape of the component being printed. These cooling rates are likely to hold tremendous bearing on resultant phase and microstructure in the component fabricated by LPBF process and hence it is important to understand and control the effect of these process and material parameters on cooling rate.

**Figure 3:**
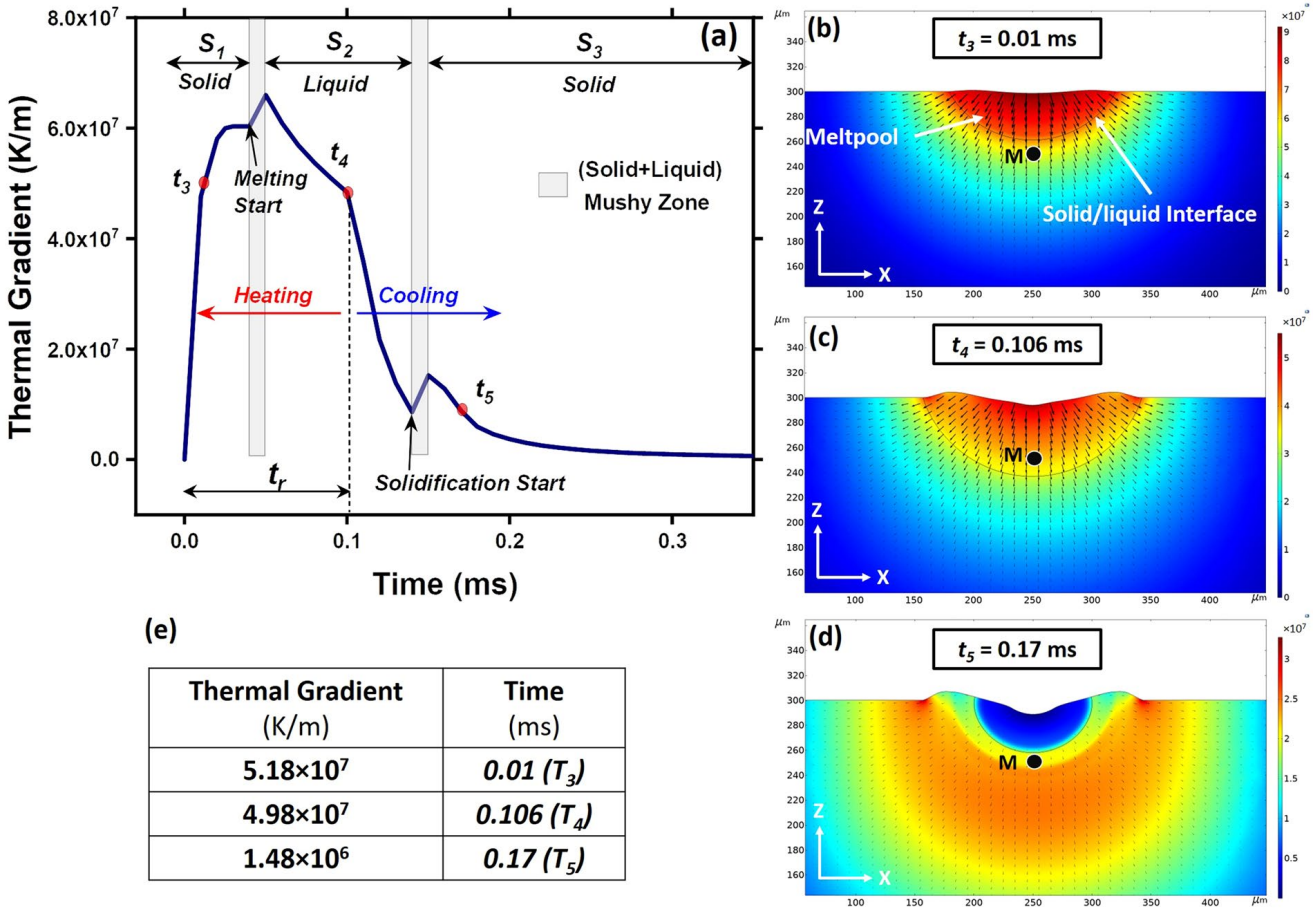
Computationally predicted (**a**) thermal gradients in solid and liquid region in asingle laser track, and (**b**–**d**) corresponding simulation of events at specific times in YZ plane with thermal gradient mapping, and (**e**) numerical thermal gradients at different events.

The magnitude and direction of the thermal gradient predominantly affect the grain morphology, grain growth direction, and crystallographic direction. In addition, a magnitude of the thermal gradient in both solid and liquid regions affects the stability of the solid-liquid interface. Thus, the thermal gradient at a given location M was monitored as a function of time under the influence of previously simulated (Fig. 2a–e) single laser track and presented in Fig. 3a. The fixed position of the location M during evolution of meltpool can be noticed in Fig. 3(b–d), which in addition represents the evolution of the thermal gradient in both meltpool and solid region. The arrows simulated in the image denote the direction of heat dissipation/heat flow (*q*′), which is opposite to the direction of thermal gradient ($G$). The length of the arrow represents the magnitude of the thermal gradient. Additionally, computationally predicted magnitudes of thermal gradients at location M at distinct events ${t}_{3}$, ${t}_{4}$, and ${t}_{5}$ are provided in the table of Fig. 3e. These specific times were selected to understand the evolution of thermal gradient at location M while it undergoes different physical states (solid, liquid, mushy).

The variation of thermal gradient as function of time at location M can be distinctly divided into three segments ${S}_{1}$, ${S}_{2}$, and ${S}_{3}$ associated with the sequential phase transitions of material at that location (Fig. 3a). In segment ${S}_{1}$, location M remains in solid-state and initially experiences a steep rise in the thermal gradient during heating due to increase in the temperature difference Δ*T* between location M and neighboring locations (Fig. 3a,b). As the incoming solid-liquid interface approaches location M, Δ*T* continue to increase at substantially reduced rate until beginning of melting at this location, which is reflected in the gradual change in slope of thermal gradient versus temperature curve in Fig. 3a. This may be due local heat loss experienced by location M due to latent heat absorbed by the approaching solid-liquid interface from surrounding region. The change in the slope of the thermal gradient can be seen as location M enters a mushy state, which possesses a distinct set of thermophysical properties over a small region and thus, affecting the rate of change of thermal gradient (Fig. 3a). When location M is in completely liquid state in meltpool (Fig. 3c), it experiences continuous drop in thermal gradient at specific rate till the end of residence time ${t}_{r}$ or ${t}_{4}$, where it reaches the peak temperature. This continuous drop in thermal gradient is mainly due to the high thermal conductivity of liquid Ti6Al4V and change in other thermophysical properties of liquid state compared to mushy and solid state of the material. In addition, continuous increase in the size of meltpool until the end of residence time also causes a drop in the thermal gradient. In remaining section of segment ${S}_{2}$ (after the end of laser residence time) the meltpool begins to cool and hence the peak temperature of the liquid material at the location M rapidly drops, thereby continuously and rapidly reducing the thermal gradient at that location until it reaches the solidification temperature. As the solidification commences through the mushy zone, again rise of the thermal gradient is observed until the transformation of entire volume of liquid at location M into solid. Segment ${S}_{2}$ is followed by segment ${S}_{3}$ representing the solid state of the material at location M (Fig. 3d). In segment ${S}_{3}$, the solid slowly cools and hence thermal gradients drops gradually.

The computational prediction of thermal gradient provides an insight into a fundamental aspect of variation of thermal gradient in the vicinity of solid-liquid interface during heating and cooling events. During heating, the thermal gradient in the solid region ahead of solid-liquid interface remains lower than that in the liquid region (Fig. 3a–c). Thus, the solid-liquid interface is highly unstable and solidification does not occur. On the other hand, as the solid region ahead of the solid-liquid interface experiences a higher thermal gradient than that in the liquid region (Fig. 3a,d), the solidification proceeds with a stable solid-liquid interface.

#### Thermal Evolution in Single Layer

**Figure 4:**
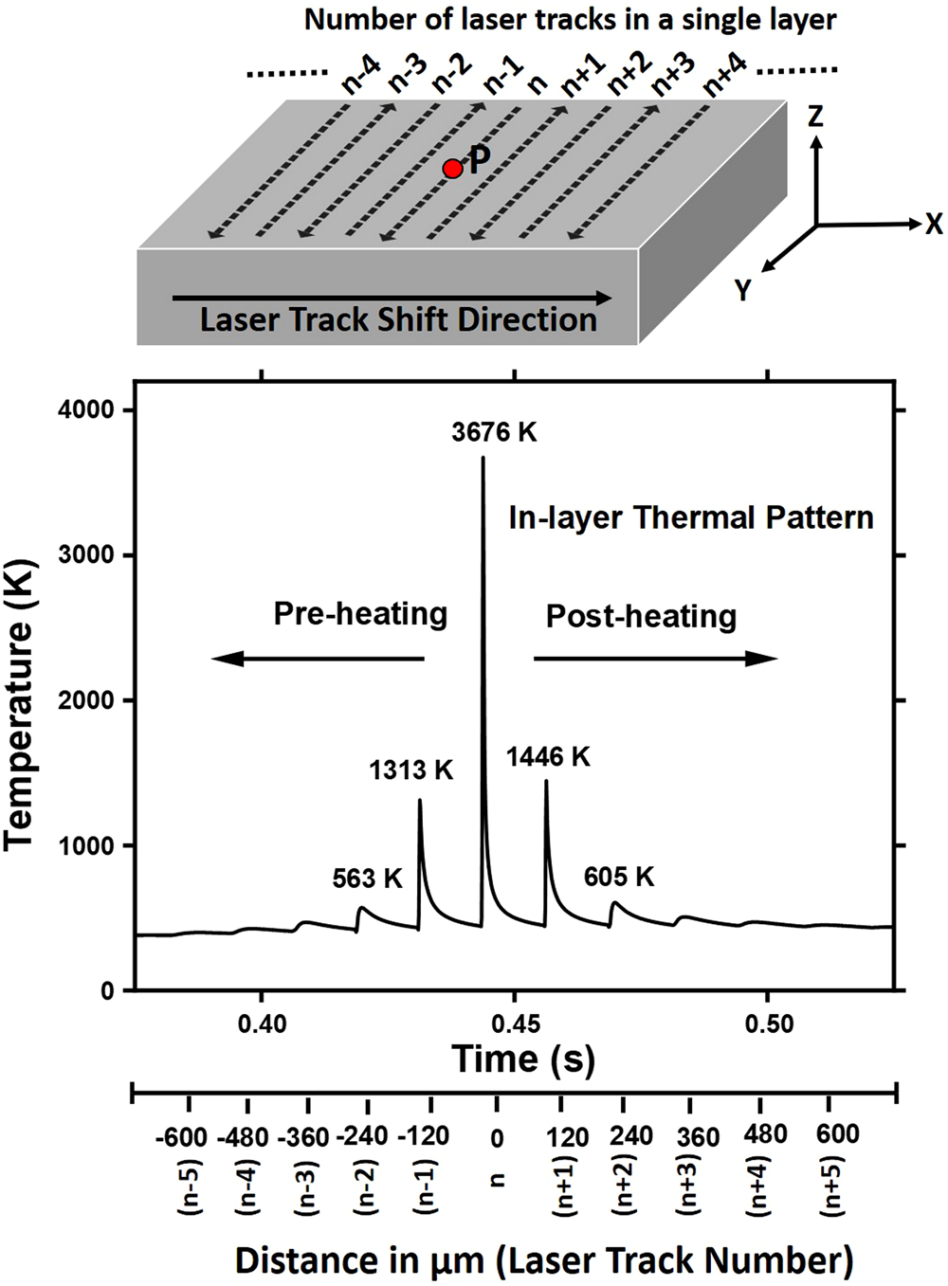
Computationally predicted in-layer thermal pattern experienced by location P in a layer.

In order to seek an insight into the thermal history (evolution) of a single layer during LPBF process, a computational simulation was performed employing the multi-physics FEM model developed during this study and described earlier. The model took into account the architectural characteristics associated with fabrication of each layer (XY plane in Fig. 1) using LPBF process such as a) multiple laser tracks, b) bi-directional linearly parallel laser tracks, and c) constant distance between centers of two consecutive laser tracks (constant hatch spacing) as described in earlier section and depicted in Fig. 1b. In such single layer, the temperature was computationally monitored as function of time at one specific location P in the layer (XY plane) to specifically identify the preheating and post heating effects due to multi-laser track (— -, $(n-\mathrm{4)}$, $(n-\mathrm{3)}$, $(n-\mathrm{2)}$, $(n-\mathrm{1)}$, $n$, $(n+\mathrm{1)}$, $(n+\mathrm{2)}$, $(n+\mathrm{3)}$, $(n+\mathrm{4)}$, — -) process over the entire layer domain (Fig. 4). Figure 4 also provides distance on X-axis of layer domain representing the location of laser track separated by the hatch spacing (120 μm) and temperature evolution in the neighboring region to the laser track.

When the laser beam scans over *n*^*th*^ track and passes over location P, its peak temperature reaches to 3676 K (Fig. 4). Also, the prior neighboring laser tracks individually contribute to rise in the temperature of location P (Fig. 4) due to heat transfer from these laser tracks at the extents proportional to their relative distances from location P. Under the set of materials and processing parameters used in the present study, it appeared that only four prior immediate neighboring laser tracks ($(n-\mathrm{1)}$, $(n-\mathrm{2)}$, $(n-\mathrm{3)}$, and $(n-\mathrm{4)}$) up to distance of ~500 μm affect temperature at location P in the range of 425 K–1300 K and significantly contribute to temperature rise (3676 K) by *n*^*th*^ laser track. Preheating due to remaining prior laser tracks (located at a distance >500 μm) are marginal (<400 K) for any microstructural effects (Fig. 4). Such a pre-heat treatment may affect the temperature-dependent thermophysical properties of the pre-heated bed before it is melted. Furthermore, as the peak temperature at location P reaches 1313 K (>${T}_{\beta }$) due to ${(n-\mathrm{1)}}^{th}$ laser track, martensite formation may occur due to rapid cooling rate (10^5–6^ K/s, Fig. 2e). This starting microstructure is likely to affect the martensite start temperature location P during laser scanning over *n*^*th*^ track.

Similarly, laser treatment (up to *n*^*th*^ laser track) at location P follows a post-heat treatment in the range of 1446 K–471 K due to $(n+\mathrm{1)}$, $(n+\mathrm{2)}$, $(n+\mathrm{3)}$, and $(n+\mathrm{4)}$ laser tracks (Fig. 4). This post heat treatment cycle involved gradually reduced cooling rate compared to rate of cooling after *n*^*th*^ laser scan. Such a post-heating pattern may be partially conducive to reducing high residual stresses generated at location P during *n*^*th*^ laser scan.

**Figure 5:**
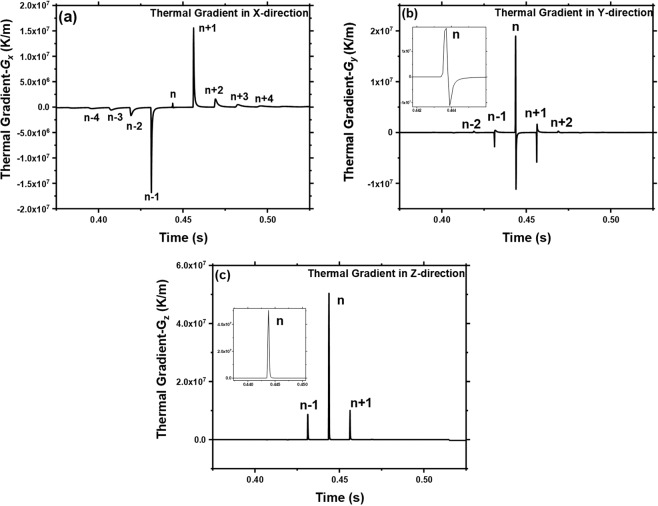
Computationally predicted thermal gradient in (**a**) X, (**b**) Y, and (**c**) Z directions at location P.

**Figure 6:**
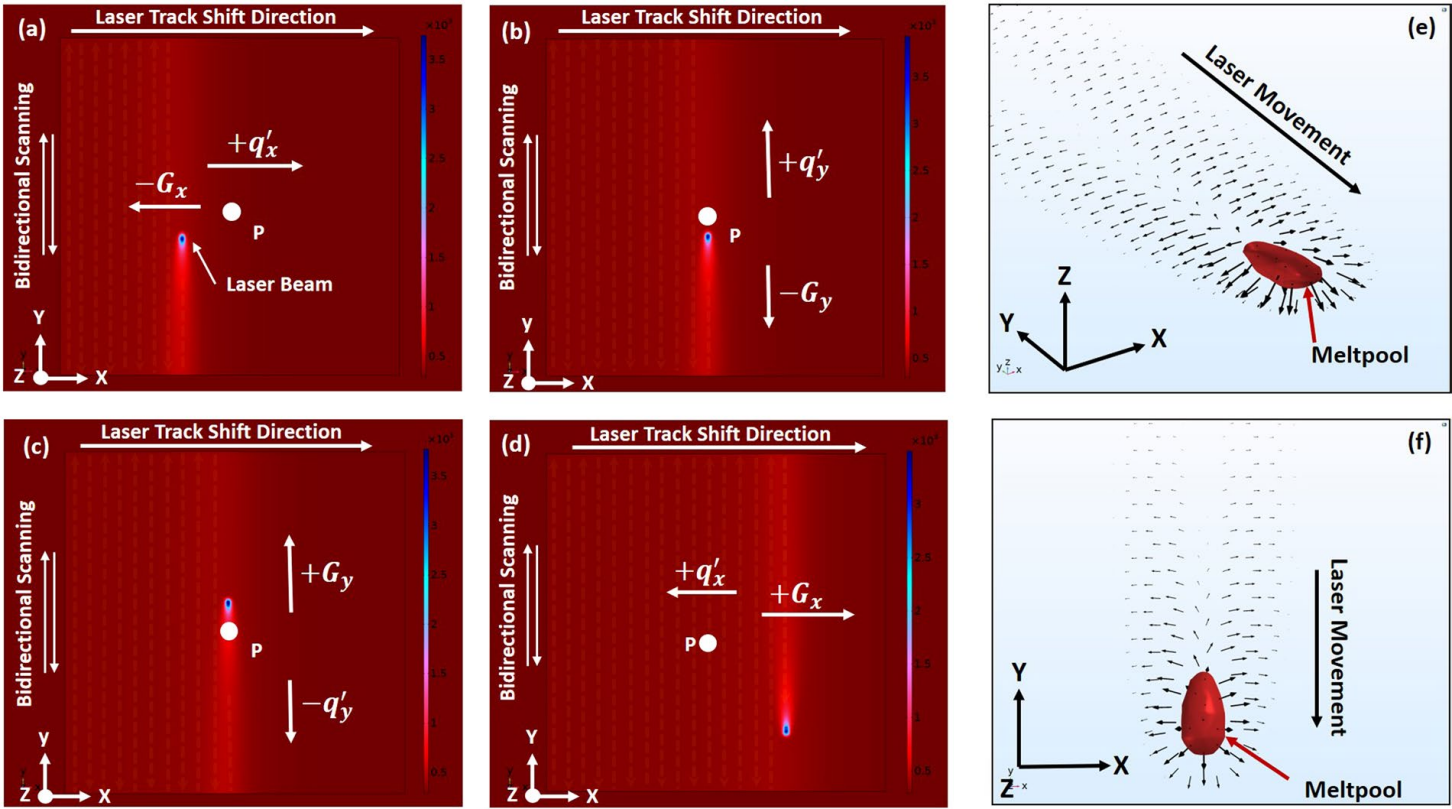
(**a**–**d**) Simulated events of laser beam scanning of layer domain depicting the direction of thermal gradient and heat transfer. (**a**) laser beam on a track to left of position P, (**b**) laser beam behind position P on same track, (**c**) laser beam ahead position P on same track, (**d**) laser beam on a track to right of position P. (**e**,**f**) Computationally simulated 3D views of the spatial distribution of heat transfer around moving meltpool.

As discussed earlier, the direction and magnitude of the thermal gradient are likely to influence the direction of grain growth, grain morphology, and crystallographic texture. In order to understand the development of microstructure and crystallographic texture in LPBF process, it is critical to study the magnitudes of thermal gradients in different directions. Thus, again the location P in layer domain was probed to obtain thermal gradients in X, Y, and Z directions with the same time frame (as in Fig. 4). These thermal gradients ${G}_{x}$, ${G}_{y}$, and ${G}_{z}$ along X, Y, and Z, respectively, as a function of time, are presented in Fig. 5a–c. It is noteworthy that these temperature gradients are computed considering the evaporative heat flux condition. Thus, the maximum temperature was the vaporization temperature and not the peak temperature achieved (>${T}_{v}$) at location P. This avoided the overestimation of the thermal gradients. From Fig. 5a–c, it was realized that at location P, the components ${G}_{x}$ and ${G}_{y}$ have a positive or negative thermal gradient values, whereas ${G}_{z}$ remains positive. The negative values of ${G}_{x}$ and ${G}_{y}$ are not associated with the undercooling effect. On the contrary, they are affected by heat flux associated with the neighboring prior and post laser tracks and their relative position with respect to location P. This concept can be more clearly realized from computationally simulated events of the laser track movement with respect to location P in the layer domain (Fig. 6a–f).

As stated earlier, the thermal gradient ($G$) is opposite in direction to heat transfer (*q*′). When the laser beam position is to the left of location P (Fig. 6a) along X-axis, the thermal gradient ${G}_{x}$ at location P will be directed towards negative X direction. Thus, ${G}_{x}$ remains negative and increases in magnitude with each laser track approaching location P (Figs. 5a and 6a). A laser track closest to location P (on its left ${(n-\mathrm{1)}}^{th}$ track) is likely to generate the highest thermal ${G}_{x}$ in the negative X-direction (Fig. 5a). When the ${n}^{th}$ track passes over location P, it being in the center of the meltpool, the magnitude of ${G}_{z}$ is highest at the instance when the laser beam lies exactly above it (Fig. 5c). As the laser tracks are aligned in Y-direction, the ${n}^{th}$ laser track while passing over location P, reverses the direction of the thermal gradient (${G}_{y}$) along Y-direction (Fig. 6b,c). The state of the thermal gradient corresponding to the laser track moving away from the location P (Fig. 6d) is a reverse mirror image of the laser track approaching location P (Fig. 6a). However, the magnitude of the thermal gradient depends on the thermal conductivity of the material and the dimension of the LPBF built part at that instance, which acts as a heat sink. The fabricated volume of the LPBF part possesses high thermal conductivity due to its higher density and being at elevated temperature compared to the volume of unprocessed powder bed.

The magnitude of thermal gradients (${G}_{x}$, ${G}_{y}$, and ${G}_{z}$) at location P decreases significantly by orders of magnitude in post heating cycle due to $n+1$, $n+2$, .. laser tracks (Fig. 5a–c). As clearly indicated in Fig. 5a–c, the thermal gradient at a given location (P) within the laser track (n-track) have distinctly different signatures in orthogonally oriented three directions (X, Y, and Z). As the thermal gradient is a primary cause for the generation of residual thermal stress, the magnitude and components of the resultant thermal gradient are also likely to wield proportionately similar signature for the residual thermal stress generated at any given location. However, these thermal gradient signatures and corresponding residual thermal stresses are likely to further evolve during subsequent post-treatment laser tracks (n − 1, n + 2, ..... etc.) in previously treated laser tracks (n, n − 1, n − 2, .... etc.) due to the thermal annealing effect. Although in the present work thermal stresses are not evaluated experimentally and computationally, separate parallel efforts to evaluate them are underway and the outcome of these efforts will be reported separately in due course of time. Furthermore, the computational prediction of thermal gradient at a given location and desired temperature assists in mapping physical orientation of grain and phase morphology at that location and temperature. However, these thermal gradients (${G}_{x}$, ${G}_{y}$, and ${G}_{z}$) are likely to change as the new layer is built on the top of current layer. Hence, based on current computational model, a thermal evolution in multilayer volume is described in the following section.

#### Thermal Evolution in Multilayer Structure

**Figure 7:**
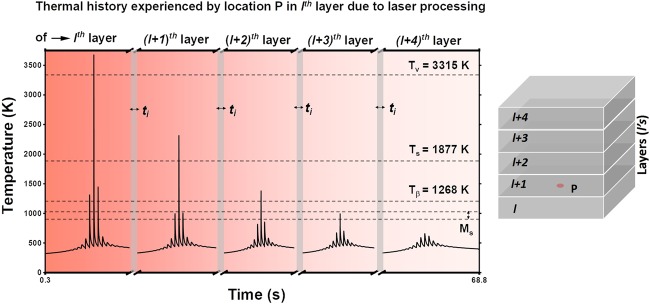
Overall thermal history experienced by location P on the surface of *l*^*th*^ layer due to fabrication of subsequent layers on *l* layer.

The temperature at location P in ${l}^{th}$ layer was computationally probed while subsequent layers were added in Z-direction and thermal influence of these subsequent layers was predicted at location P (Fig. 7). While subsequent layers are built, location P on the surface of ${l}^{th}$ layer experiences similar in-layer thermal patterns but at reduced intensity compared to the thermal pattern experienced during fabrication of ${l}^{th}$ layer (Fig. 6). The building of ${(l+\mathrm{1)}}^{th}$ layer again thermally treat location P in ${l}^{th}$ layer with a similar pattern of preheating-melting-post-heating with relatively lower peak temperatures due to individual laser tracks. The remelting of location P while building of ${(l+\mathrm{1)}}^{th}$ layer on layer $l$, is generally intentionally achieved to generate a metallurgically sound interface between consecutive build layers during LPBF process.

Under the processing conditions employed during present LPBF process and due to a substantially long interlayer delay time (*t*_*i*_ = 16 s), the heat build-up amongst the built layers is significantly low as indicated by the base line of the time-temperature relationship (Fig. 7). Furthermore, during fabrication of ${(l+\mathrm{2)}}^{th}$, layer the temperature of location P rises up to 1397 K, which is below the solidus temperature (1877 K) and above the $\beta$ transus temperature of Ti6Al4V. In the fourth thermal pattern during fabrication of ${(l+\mathrm{3)}}^{th}$ layer, the peak temperature at location P reaches up to 992 K. This temperature lies in the martensitic start (${M}_{s}$) temperature range (848 K–1048 K) of Ti6Al4V reported in the literature^22^. Eventually, during fabrication of ${(l+\mathrm{4)}}^{th}$ layer, the temperature of location P barely reaches up to 670 K. Although during fabrication of subsequent layers the peak temperatures at location P gradually and substantially drop, the thermal cycles follows the same trend of the in-layer thermal pattern. The overall cyclic thermal evolution experienced by location P during LPBF fabrication is likely to substantially influence microstructural and phase evolution along with the state of stress in the component. The following section describes the evolution of microstructure, phase, and crystallographic texture during present work and the authors intend to provide work on evolution of stress during LPBF process in a separate report due to the extensive nature of the subject matter.

### Microstructural evolution

**Figure 8:**
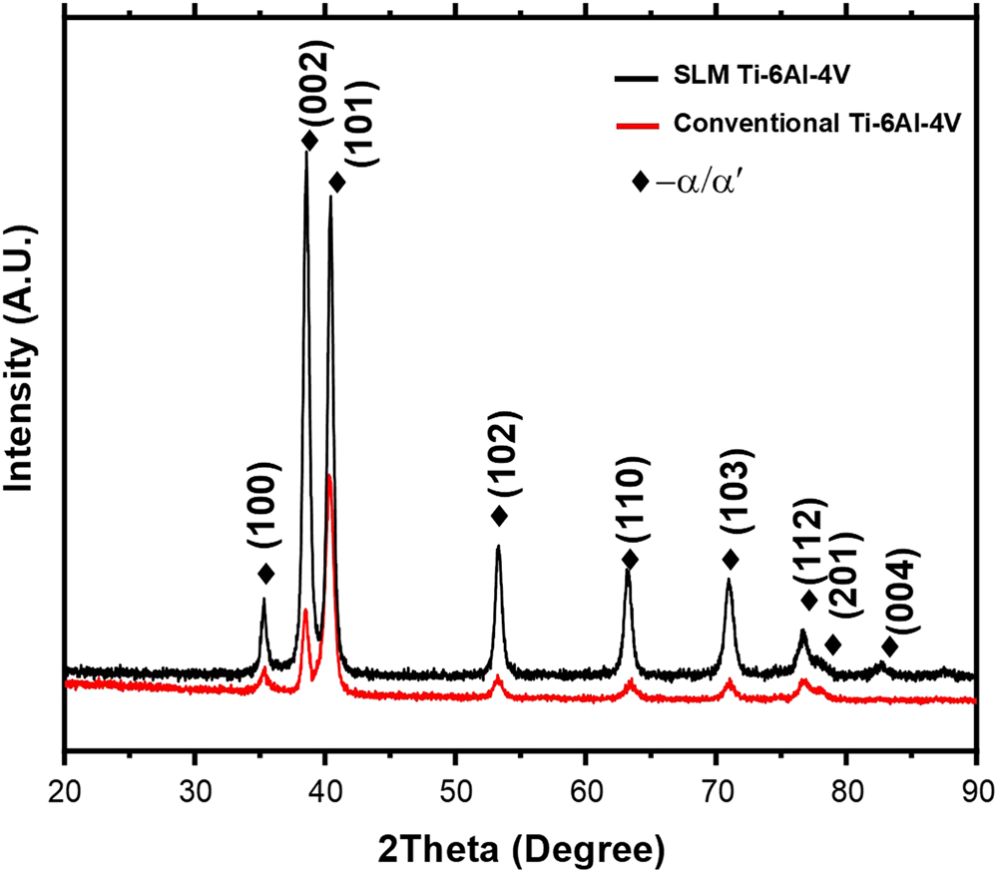
X-ray diffraction spectra of LPBF printed and conventionally cast Ti6Al4V alloy.

In Ti6Al4V alloy, the cooling rate exceeding 410 K/s from above martensitic start temperature (850 K–1078 K) yields martensitic *α*′ phase (HCP)^22^. Based on the thermal history predicted in Fig. 7 and a predicted cooling rate in the range of 10^5^–10^6^ K/s in the given ${M}_{s}$ range, probability of formation of *α*′ phase is high. The X-ray diffraction profile of LPBF printed Ti6Al4V indicated the presence of hexagonally packed (HCP) phase, which may corresponds to either *α*′ or $\alpha$ (Fig. 3) since, they share the same crystallographic structure (HCP). However, due to the supersaturated nature of *α*′ phase the lattice parameters are slightly stretched. Thus, based on XRD spectra the lattice parameters were calculated as $a$= 2.939 ± 0.0009 Å and $c$ = 4.672 ± 0.0012 Å, which coincide well with previously reported parameters of *α*′^13,14^. In addition, the much stronger intensity of the (002) Braggs peak compared to the conventionally cast Ti6Al4V alloy is noticeable (Fig. 8). This indicates that the majority of grains/phase in build plane (XY) are oriented along [002] direction. Such a strong texture may have been due to preferentially growing direction of prior $\beta$ grains in <001> direction^22,23^ and the *α*′/$\alpha$ texture is inherited from $\beta$ grain along <001> direction^23^. Such phase transition via inheritance of the crystallographic texture from prior $\beta$ grain to *α*′/$\alpha$ can be a result of inadequate diffusion and homogenization of compositional species due to extremely rapid cooling rates (>${10}^{5}$ K/s) associated with the LPBF process. Similar transitions have been previously reported in laser processed ferrous material systems^24–26^.

**Figure 9:**
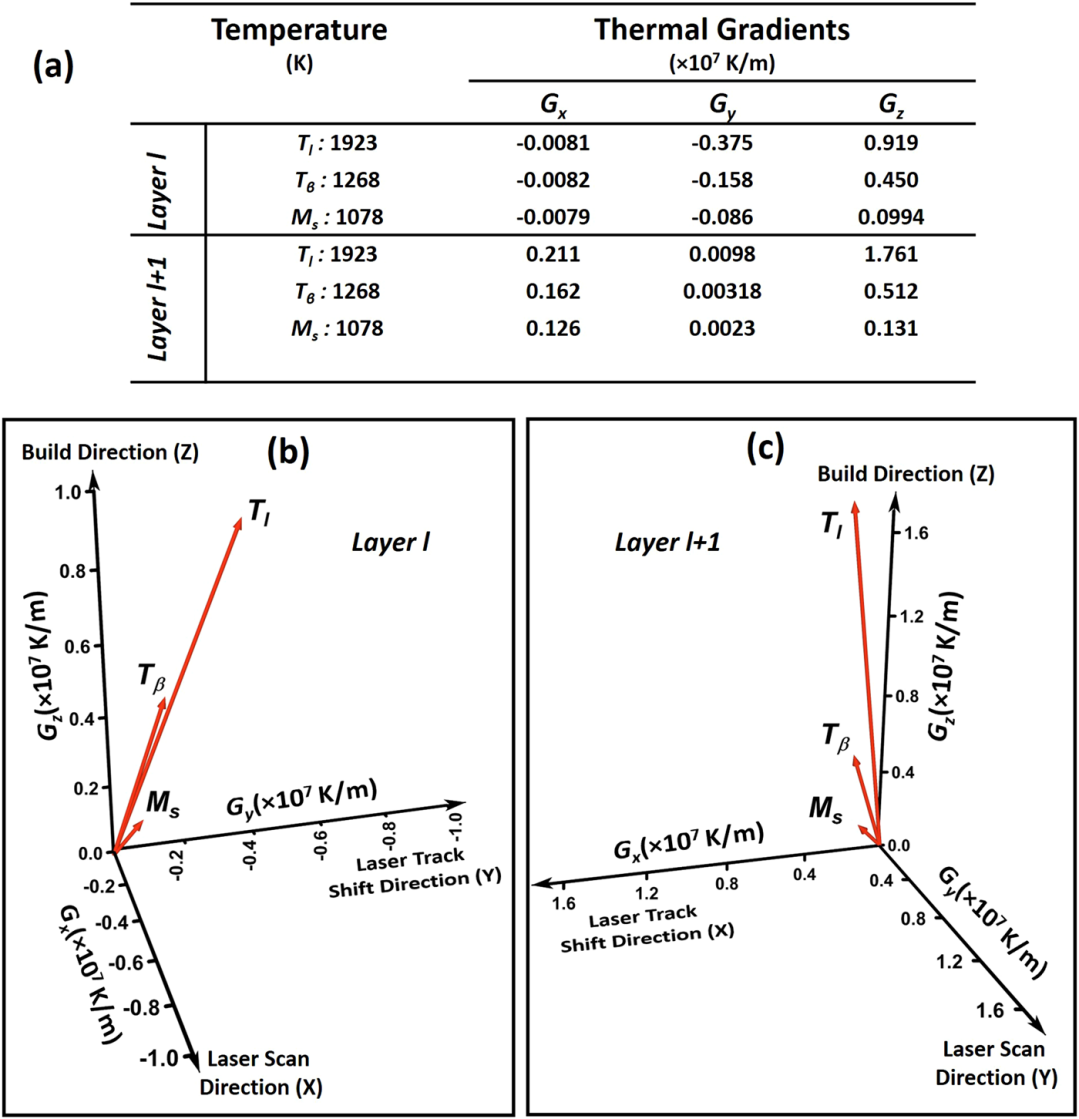
Computationally predicted thermal gradients at location P during building of layer $l$ and layer $(l+\mathrm{1)}$ (**a**) numerical values of individual components of thermal gradients ${G}_{x}$, ${G}_{y}$, and ${G}_{z}$ in layer $l$ and layer $(l+\mathrm{1)}$ and (**b**,**c**) graphical representation of resultant thermal gradient vectors at ${T}_{l}$, ${T}_{\beta }$, and ${M}_{s}$ in layer $l$ and layer $(l+\mathrm{1)}$.

**Figure 10:**
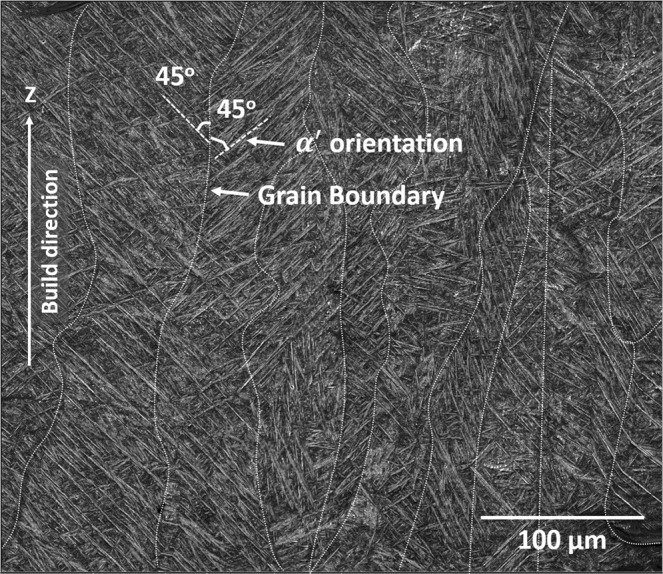
SEM micrograph of LPBF printed Ti6Al4V alloy along direction in YZ plane.

Since the remelting of location P occurs during fabrication of layer $(l+\mathrm{1)}$ on layer $l$, the grain morphology and crystallographic direction is likely to be influenced by the magnitude and direction of thermal gradient at given time and temperature at location P. Thus, vector components (along X, Y, and Z) of thermal gradients (${G}_{x}$, ${G}_{y}$, and ${G}_{z}$) at location P were computationally monitored at different transformation temperatures during fabrication of both $l$ and $(l+\mathrm{1)}$ layers and corresponding resultant three-dimensional vectors of thermal gradient at various transformation temperatures are presented in Fig. 9. During fabrication of layer $l$ when temperature at location P reaches the liquidus temperature (1923 K) of Ti6Al4V, the resultant thermal gradient is mainly influenced by ${G}_{z}$ (Fig. 9a,b). At this stage, location P experiences inclination of resultant thermal gradient towards -Y direction which is laser track shift direction. Similarly during fabrication of layer $(l+\mathrm{1)}$, the resultant thermal gradient is strongly influenced by ${G}_{z}$ (Fig. 9a,c) and it is more so compared to that during fabrication of layer $l$ (Fig. 9a). The variation in position and the distance of laser heat source from location P during fabrication of layers $l$ and $(l+\mathrm{1)}$ tend to influence the resultant direction and magnitude of thermal gradient. Similar directional orientation of resultant thermal gradient was computationally predicted at location P when it reaches ${T}_{\beta }$ temperature (Fig. 9a–c). Such a highly directional behavior of resultant thermal gradient towards +Z during $\beta$ grain formation is expected to have columnar prior $\beta$ grain morphology along Z direction. The micrographs of LPBF printed Ti6Al4V alloy showing columnar grain morphology of prior $\beta$ grain mainly oriented along build direction (Z-axis) in YZ plane confirmed this computational prediction (Fig. 10). However, most of the grains appeared to be longer than the layer thickness of the powder bed (30 μm), suggesting the epitaxial growth of $\beta$ grains across several layers^22^. During the fabrication of a layer in the LPBF process, the growing meltpool boundary penetrates and consequently re-melts the part of a previously fabricated layer. As this meltpool solidifies, the solidification begins at the solid/liquid interface lying in the previous layer. The solidified grains continue to follow previous layer crystallography in columnar morphology as they remain under the influence of resultant thermal gradient at that location. The width of the columnar grain is also influenced by the thermal gradients in the X and Y direction. Since the magnitude of the thermal gradient in X and Y direction is relatively lower compared to that in Z direction, the width of the columnar grain (71.1 ± 25 μm) remains lower than its height (900 ± 150 μm). Furthermore, it can be noticed that the majority of martensite plates within the prior $\beta$ grain are aligned at ±45° to build direction (Z) (Fig. 10). The martensitic growth is governed by high thermal stresses induced due to high thermal gradients (10^6^–10^7^ K/m) associated with high heating and cooling rates (10^5^–10^7^ K/s). Thus, the direction of resultant thermal gradients at location P at ${M}_{s}$ temperature during fabrication of layer $(l+\mathrm{1)}$ was predicted (Fig. 9c). This orientation when projected in XZ plane, indicated its inclination at ~45° with respect to Z axis. The locations such as P and other lying exactly above it along Z axis in subsequent build layers are likely to experience the resultant thermal gradient at ${M}_{s}$ (848 K–1049 K) aligned at ±45° to Z direction in either in XZ or YZ plane due to orthogonally built layers (Fig. 1). Such a directional thermal gradient associated with residual stresses may influence *α*′-$\beta$ crystallographic orientation selection amongst different variants to cause martensite morphology to align at ±45° with respect to build direction (Z).

**Figure 11:**
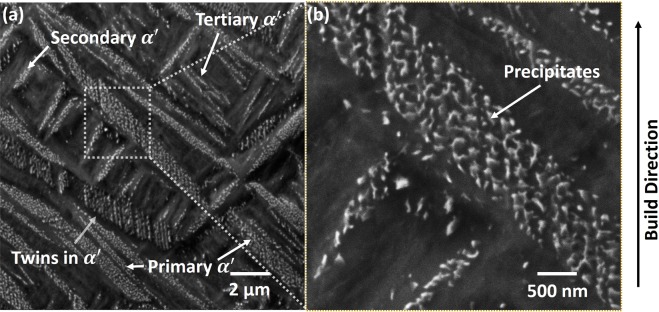
SEM micrographs of Ti6Al4V alloy along build direction in YZ plane showing (**a**) hierarchical martensitic structure and (**b**) distinct precipitate (bright) within martensite plate.

**Figure 12:**
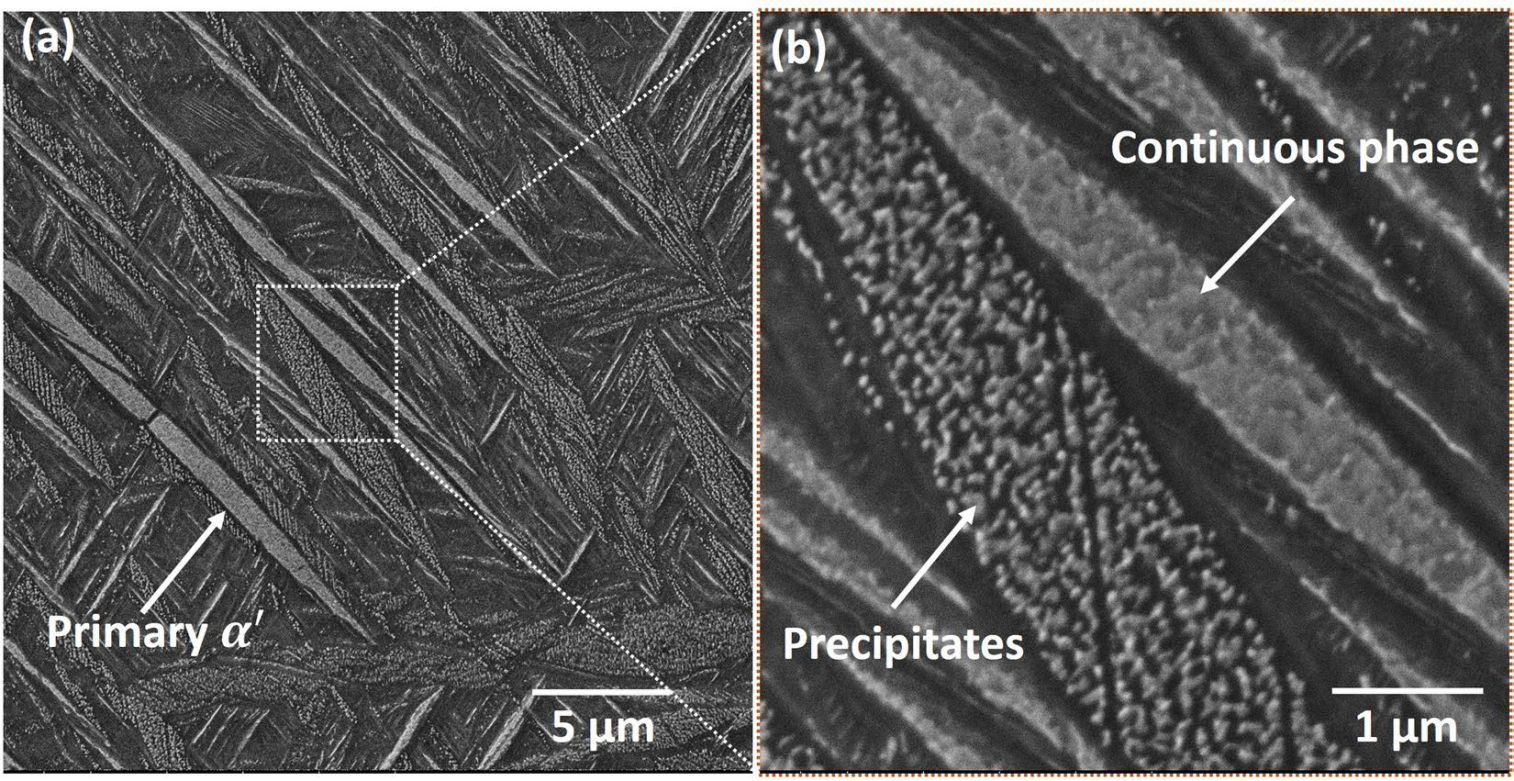
SEM micrographs of Ti6Al4V alloy along build direction in YZ plane showing morphological variation of precipitates (within martensite) amongst martensite plates (**a**) low magnification view, and (**b**) a magnified view of martensite plates.

To study the effect of thermal history on microstructural evolution in LPBF printed Ti6Al4V samples, high magnification nano SEM micrographs of the etched sample were obtained. The nano SEM micrographs collected along the build direction in YZ plane of the samples are presented in Fig. 11. The microstructure reveals a hierarchical acicular martensitic structure (Fig. 11a), similar to the previous findings^12–14^. The primary martensite (*α*′) nucleates at the columnar $\beta$ grain boundary while secondary, tertiary, and quaternary martensite (*α*′) grow orthogonal to each other^12–14^. As discussed earlier, very high cooling rates (Fig. 2) and thermal gradients (Fig. 4) in a cyclic manner (Figs. 8 and 9) are likely to induce tremendous thermal stresses during LPBF fabrication. This leads to high residual strain, which is accommodated by defects like dislocations and twins. Internal twins in martensite largely accommodate such high residual strain. In addition to the features mentioned above, the martensite plate are decorated with a distinct phase (bright) precipitated with random morphology (Fig. 11b). The EDS spot analysis of these random morphological precipitates disclosed no compositional variation within the sensitivity limits of the instrument. The samples were electropolished, followed by etching by Kroll’s etchant, which corrodes some of the alloying elements at different rates revealing the microstructural features. The Kroll’s etchant corrodes $\beta$ phase faster than $\alpha$ phase. Since the elements differentiating $\alpha$ and $\beta$ are Al and V respectively, they are likely to be the reason behind different corrosion rates of these phases. Vanadium being $\beta$ stabilizer corrodes faster. Considering this analogy, the dark region (which is more etched) in the martensite plate appears to be V rich, and the brighter phase is Al rich. Furthermore, in some martensite plates, the bright phase appeared as continuously rough morphology (Fig. 12a,b). Such a morphological appearance may arise from different orientations of these martensite plates. This suggests that the bright phase surrounds dark regions that are probably V rich. In summary, the etched microstructure indicates the compositional variation within the martensite plate. Such a compositional variation within the microstructural features is likely to occur due to multiple in-layer thermal patterns associated with the processing architecture of LPBF process. Thus, various thermal cycles generated during LPBF process lead to several $\beta$ $\to$ *α*′ $\to$ *β* $\to$ *α*′ transformation followed by gradually decreasing cyclic ageing treatment from 670 °C with rapid heating and cooling rates (Fig. 8). Such a cyclic thermal pattern inducing distinct ageing treatment is likely to allow short range diffusion of the atoms within martensitic plate, thereby leading to compositional variation for formation of packets of V rich regions. However, further detail investigation of these phases will be confirmed through transmission electron microscopy (TEM) complemented with atom probe studies and will be reported in a separate publication.

## Conclusion

The computational model developed in the current study helped to understand the thermal evolution during single laser tracks associated with the LPBF fabrication of Ti6Al4V alloy. The cooling rate (10^5^–10^7^ K/s) predicted at martensite start temperature supported formation of martensite as confirmed through XRD and SEM micrographs. The computationally predicted thermal evolution during processing a single layer yielded thermal history with varying cooling rates of the order of 10^5^–10^7^ K/s and directional thermal gradients of magnitude 10^6^–10^7^ K/m. Furthermore, the thermal evolution in multilayer LPBF part was computationally obtained to study the thermal history experienced by multilayer LPBF part. The corresponding direction and magnitude of various components (${G}_{x}$, ${G}_{y}$, and ${G}_{z}$) of thermal gradient and its resultant were predicted. These computational results assisted in understanding the experimental observations such as formation of martensite phase, columnar prior $\beta$ grains, ±45° morphological orientation of martensite. The compositional variation occurring within the martensite plate was analyzed and understood using predicted thermal history experience by certain region in a layer.
